# Association of dietary carbohydrate intake with risk of mortality in maintenance hemodialysis patients: a multicenter prospective cohort study

**DOI:** 10.1093/ckj/sfaf124

**Published:** 2025-04-28

**Authors:** Qiuxia Zhong, Zizhen Lin, Yaya Yang, Yan Huang, Xiaolei Lan, Chaoying Xia, Yaozhong Kong, Qijun Wan, Yumin Li, Sheng Huang, Yan Liu, Aiqun Liu, Fanna Liu, Xianhui Qin, Youbao Li, Min Liang

**Affiliations:** National Clinical Research Center for Kidney Disease, National Key Laboratory for Prevention and Treatment of Multi-organ Injury, Guangdong Provincial Clinical Research Center for Kidney Disease, Division of Nephrology, Nanfang Hospital, Southern Medical University, Guangzhou, China; National Clinical Research Center for Kidney Disease, National Key Laboratory for Prevention and Treatment of Multi-organ Injury, Guangdong Provincial Clinical Research Center for Kidney Disease, Division of Nephrology, Nanfang Hospital, Southern Medical University, Guangzhou, China; National Clinical Research Center for Kidney Disease, National Key Laboratory for Prevention and Treatment of Multi-organ Injury, Guangdong Provincial Clinical Research Center for Kidney Disease, Division of Nephrology, Nanfang Hospital, Southern Medical University, Guangzhou, China; National Clinical Research Center for Kidney Disease, National Key Laboratory for Prevention and Treatment of Multi-organ Injury, Guangdong Provincial Clinical Research Center for Kidney Disease, Division of Nephrology, Nanfang Hospital, Southern Medical University, Guangzhou, China; National Clinical Research Center for Kidney Disease, National Key Laboratory for Prevention and Treatment of Multi-organ Injury, Guangdong Provincial Clinical Research Center for Kidney Disease, Division of Nephrology, Nanfang Hospital, Southern Medical University, Guangzhou, China; National Clinical Research Center for Kidney Disease, National Key Laboratory for Prevention and Treatment of Multi-organ Injury, Guangdong Provincial Clinical Research Center for Kidney Disease, Division of Nephrology, Nanfang Hospital, Southern Medical University, Guangzhou, China; Nephrology Department, The First People's Hospital of Foshan, Foshan, China; Nephrology Department, Shenzhen Second People's Hospital, Shenzhen, China; Nephrology Department, Huadu District People's Hospital of Guangzhou, Guangzhou, China; Nephrology Department, Southern Medical University Affiliated Nanhai Hospital, Foshan, China; Nephrology Department, Guangzhou Red Cross Hospital, Medical College of Jinan University, Guangzhou, China; Nephrology Department, The Third Affiliated Hospital of Southern Medical University, Guangzhou, China; Nephrology Department, Jinan University First Affiliated Hospital, Guangzhou, China; National Clinical Research Center for Kidney Disease, National Key Laboratory for Prevention and Treatment of Multi-organ Injury, Guangdong Provincial Clinical Research Center for Kidney Disease, Division of Nephrology, Nanfang Hospital, Southern Medical University, Guangzhou, China; National Clinical Research Center for Kidney Disease, National Key Laboratory for Prevention and Treatment of Multi-organ Injury, Guangdong Provincial Clinical Research Center for Kidney Disease, Division of Nephrology, Nanfang Hospital, Southern Medical University, Guangzhou, China; National Clinical Research Center for Kidney Disease, National Key Laboratory for Prevention and Treatment of Multi-organ Injury, Guangdong Provincial Clinical Research Center for Kidney Disease, Division of Nephrology, Nanfang Hospital, Southern Medical University, Guangzhou, China

**Keywords:** dietary carbohydrate intake, maintenance hemodialysis, mortality

## Abstract

**Background:**

Current evidence on the relationship between dietary carbohydrate intake (DCI) and mortality risk among patients undergoing maintenance hemodialysis (MHD) is limited. Moreover, the joint impact of DCI and dietary energy intake (DEI) on mortality remains unclear. Therefore, we aimed to investigate both the individual and combined associations of DCI and DEI with all-cause and cardiovascular disease (CVD) mortality.

**Methods:**

This study included 1044 MHD patients from eight outpatient dialysis centers across China. The DCI, expressed as a percentage of carbohydrate intake in total energy intake, was determined via 24-h dietary recalls over 3 days. The study outcomes included all-cause and CVD mortality. Cox proportional hazard models were utilized to evaluate both the individual and combined associations of DCI and DEI with mortality risk.

**Results:**

During a median follow-up of 45.6 months, 352 deaths were recorded, of which 206 (58.5%) were due to CVD. When DCIs were assessed as quartiles, patients in the fourth quartile (≥72.1%) were associated with a greater risk of all-cause mortality [hazard ratio (HR) 2.16; 95% confidence interval (CI) 1.10, 4.25] than patients in the first quartile (<61.5%), whereas patients in the second quartile (61.5%–66.7%; HR 1.27; 95% CI 0.87, 1.87) and the third quartile (66.7%–72.1%; HR 1.40; 95% CI 0.84, 2.31) were not significantly different. A similar trend was found for CVD mortality. When analyzed jointly, patients with high DCIs (≥72.1%) and low DEIs (<25 kcal/kg/day) had the highest risk of all-cause and CVD mortality.

**Conclusions:**

A higher DCI was associated with a higher risk of all-cause and CVD mortality in MHD patients. Patients with high DCIs and low DEIs had a worse survival prognosis.

## Graphical Abstract

**Graphical Abstract:**
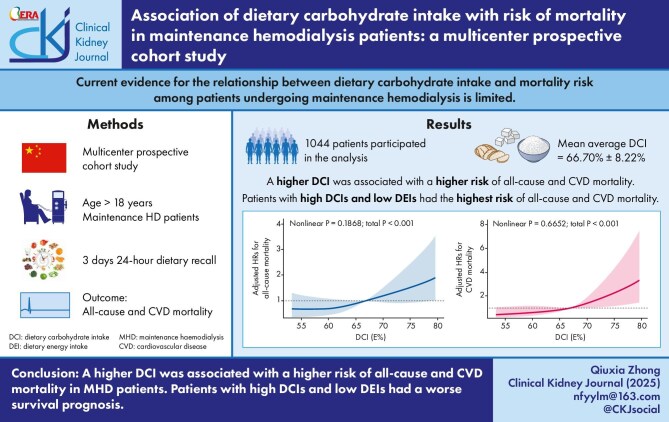

## INTRODUCTION

Globally, the number of patients with end-stage renal disease undergoing maintenance dialysis is large and increasing [1]. Cardiovascular disease (CVD) is a common complication and remains the leading cause of mortality in individuals undergoing maintenance hemodialysis (MHD). In this specific population, the incidence of CVD death is 20 times higher than that in the general population, with CVD affecting the majority of patients with MHD [2]. Therefore, it is essential to identify potentially modifiable mortality risk factors to improve patient outcomes.

Dietary carbohydrates, as the main substrate of human energy metabolism, are closely related to health. On one hand, excessive dietary carbohydrate intake (DCI) can increase serum glucose and lipid levels, which in the long term leads to obesity and diabetes mellitus [3, 4]; on the other hand, chronically low DCI can activate inflammatory pathways and promote oxidative stress [5].

Previous studies on the relationships between DCI and all-cause and CVD mortality have been controversial [6–12]. Dietary composition and nutritional strategies play a nonnegligible role in the management of patients with MHD [13]. In the setting of an impaired glomerular filtration rate, inadequate nutritional intake has a significant impact on residual renal function. Proper nutritional intake can effectively delay the progression of kidney disease. However, the relationship between DCI and the risk of mortality, particularly in patients with MHD, remains unclear. To address these limitations, this study aimed to investigate the associations between DCI and all-cause and CVD mortality in MHD patients while exploring potential effect modifications to provide comprehensive guidance for adequate and balanced diets in MHD patients.

## MATERIALS AND METHODS

### Study design and participants

The design of this study and some of the findings have been published previously [14, 15]. The study was a multicenter, prospective cohort study conducted from January 2014 to December 2015 in eight outpatient dialysis centers (Nanfang Hospital, the First People's Hospital of Foshan, Huadu District People's Hospital of Guangzhou, Guangzhou Red Cross Hospital, Guangzhou Overseas Chinese Hospital, the Third Affiliated Hospital of Southern Medical University, Nanhai District People's Hospital of Foshan and the Second People's Hospital of Shenzhen) in the Guangdong, China.

The inclusion criteria were patients over 18 years of age, who were on MHD for more than 3 months, and who had normal oral dietary intake. The exclusion criteria included hyperthyroidism, acute infection, liver cirrhosis, multiorgan failure, severe gastrointestinal disease, cognitive impairment and advanced malignancy.

Participants were followed up at each dialysis session, during which possible endpoint events and vital signs were recorded by physicians and trained researchers. The study was approved by the Medical Ethics Committee of Nanfang Hospital, and all participants signed an informed consent form before enrollment.

### Data collection and measurements

Baseline data, including sociodemographic characteristics, medical history, medication use and lifestyle information, were collected by trained researchers via standard operating procedures, and each participant was interviewed via a standardized questionnaire designed specifically for this study. Diabetes mellitus was defined in this study as a previous history of diabetes mellitus or the use of glucose-lowering therapy, including insulin or oral hypoglycemic agents. Hypertension was defined as a prior history of hypertension or the use of antihypertensive drugs. A history of CVD was defined as a history of congestive heart failure, angina pectoris, myocardial infarction, transient ischemic attack or cerebrovascular accident, and peripheral arterial disease. Patients with a recent history of smoking and alcohol consumption were defined as current smoking and drinking, respectively.

The baseline physical examination was performed after dialysis, and the dry weight of the patient was estimated. Blood samples were taken before hemodialysis. Biochemical parameters, including serum albumin, blood urea nitrogen (BUN), C-reactive protein (CRP), triglycerides, total cholesterol, calcium and phosphate, were measured according to the standardized protocol at each dialysis center. The urea kinetic model formula was used to calculate Kt/V = –ln(post-BUN/pre-BUN – 0.008 × *t*) + (4 – 3.5 × post-BUN/pre-BUN) × UF/post weight, where UF is the ultrafiltration volume (in liters) and *t* is the dialysis time.

Dietary intake was evaluated by trained investigators via the automated multiple-pass method. This included a 24-h dietary recall performed on three days (two nondialysis days and one dialysis day) within 1 week. Twenty-four-hour dietary recall is a relatively rapid assessment method that systematically obtains recent information about food intake and is widely used in epidemiological studies [16, 17]. The automated multiple-pass method was developed by the US Department of Agriculture and employs a five-step, multiple-pass, computer-based, interviewer-managed program to obtain dietary reviews. Further details on this assessment method can be found in another publication [18].

Nutrient intake in this study was calculated via dietary software, version 2.0 (Zhending), in which the nutrient model was based on the Chinese Food Composition Table developed by the Center for Disease Control and Prevention of China in 2009.

The DCI, which refers to starch and sugar intake, excluding dietary fiber, was expressed as a percentage of dietary carbohydrates in total energy intake. The participants were analyzed according to DCI quartile groups. The dietary energy intake (DEI), dietary protein intake (DPI) and dietary fat intake (DFI) by the participants was standardized by the actual dry weight of the patient and presented in terms of kcal/kg/day and g/kg/day, whereas the intake of dietary fiber was expressed as the actual intake (g/day).

### Study outcomes

All patients were followed up until death, renal transplantation, peritoneal dialysis, loss to follow-up or the end of the study in July 2019. The primary outcomes were all-cause mortality and CVD mortality. All-cause death was defined as death from any cause, whereas CVD death was defined as death from myocardial infarction, heart failure, sudden cardiac death, stroke, cardiovascular hemorrhage or other vascular causes. Evidence of death is proven through a death certificate provided by the hospital or through a consensus among experts if the death occurred outside the hospital.

### Statistical analysis

Continuous variables in demographic characteristics are expressed as mean ± standard deviations, or median (interquartile ranges), while categorical variables are expressed as counts (proportions). Baseline characteristics were compared via the χ^2^ test for categorical variables and analysis of variance tests or the Kruskal–Wallis test for continuous variables.

Univariate and multivariate Cox proportional hazard regression models [hazard ratio (HR) and 95% confidence interval (CI)] were employed to evaluate the associations between DCI and all-cause mortality and CVD mortality risk with and without adjustment for dialysis center, age, sex, comorbidities (history of CVD, hypertension, diabetes), smoking status, alcohol consumption, Kt/V, dialysis duration, body mass index, albumin, CRP, total cholesterol, calcium, phosphate, triglycerides, DEI, DFI, DPI and dietary fiber intake. In the Cox proportional hazard model, the risk period was defined as the time from study entry to death, kidney transplantation, peritoneal dialysis or the end of the most recent investigation, whichever occurred first. We also used restricted cubic splines with five knots at the 5th, 25th, 50th, 65th and 95th centiles to flexibly model the relationship between DCI and the risk of all-cause and CVD mortality.

In addition, an exploratory analysis was conducted to assess the potential impact of modifying the relationship between DCI and CVD mortality. Variables including age (<60 vs ≥60 years), sex (male vs female), diabetes status (yes vs no), history of hypertension (yes vs no), history of CVD (yes vs no), serum albumin levels (<38 g/L vs ≥38 g/L), CRP levels (<3 mg/L vs ≥3 mg/L), DEI (<25 kcal/kg/day vs ≥25 kcal/kg/day) and DPI (<1.0 g/kg/day vs ≥1.0 kcal/kg/day) were considered.

All analyses were conducted with a two-tailed *P*-value <.05 deemed statistically significant. All the statistical analyses were conducted via R software (http://www.R-project.org), version 4.3.1.

## RESULTS

### Study participants and baseline characteristics

As shown in the flowchart, a total of 1302 adult patients with MHD were enrolled in this cohort. After the exclusion of participants who were not eligible for inclusion or who were missing key variables, 1044 patients participated in the analysis (shown in Supplementary data, Fig. S1). The baseline characteristics of the participants are summarized in Table 1 according to DCI quartiles. The average age of the study population was 54.05 ± 15.10 years; 603 (57.8%) of them were male. The mean DCI was 66.70% ± 8.22%. Individuals with higher levels of DCI had lower cholesterol, BUN, serum calcium, DEI, DPI and DFI levels, and tended to be male (shown in Table 1).

**Tabel 1:** Baseline characteristics of the study participants according to DCI levels (E%).

		Categories of DCI, E%				
Characteristic	Overall	<61.5	61.5–66.7	66.7–72.1	≥72.1	*P*
*N*	1044	259	262	260	263	
Age, years	54.05 ± 15.10	54.11 ± 15.42	53.44 ± 15.49	54.62 ± 14.40	54.02 ± 15.11	.847
Male, *n* (%)	603 (57.8)	135 (52.1)	154 (58.8)	143 (55.0)	171 (65.0)	.019
Current smoking, *n* (%)	150 (14.4)	36 (13.9)	44 (16.8)	35 (13.5)	35 (13.3)	.634
Current alcohol consumption, *n* (%)	38 (3.6)	16 (6.2)	8 (3.1)	6 (2.3)	8 (3.0)	.086
Comorbid conditions						
Diabetes, *n* (%)	279 (26.7)	82 (31.7)	62 (23.7)	67 (25.8)	68 (25.9)	.195
Hypertension, *n* (%)	894 (85.6)	231 (89.2)	222 (84.7)	220 (84.6)	221 (84.0)	.308
History of CVD, *n* (%)	201 (19.3)	54 (20.8)	53 (20.2)	53 (20.4)	41 (15.6)	.381
BMI, kg/m²	21.25 ± 3.38	21.32 ± 3.38	21.15 ± 3.83	21.21 ± 3.13	21.34 ± 3.13	.909
Laboratory variables						
Albumin, g/L	38.10 ± 3.79	37.99 ± 3.63	38.17 ± 3.52	38.37 ± 3.78	37.86 ± 4.20	.452
CRP, mg/L	2.71 (1.00, 7.34)	3.06 (1.40, 7.23)	2.04 (0.85, 7.11)	2.73 (0.94, 7.21)	2.80 (0.89, 8.23)	.167
BUN, mmol/L	26.05 ± 7.14	27.22 ± 6.80	26.57 ± 7.18	25.93 ± 7.19	24.52 ± 7.12	<.001
Triglyceride, mmol/L	1.86 ± 1.37	1.89 ± 1.31	1.84 ± 1.36	1.90 ± 1.47	1.81 ± 1.36	.881
Cholesterol, mmol/L	4.14 ± 1.13	4.29 ± 1.16	4.15 ± 1.04	4.24 ± 1.08	3.90 ± 1.18	<.001
Calcium, mmol/L	2.17 ± 0.26	2.20 ± 0.25	2.19 ± 0.27	2.16 ± 0.26	2.12 ± 0.26	.004
Phosphorus, mmol/L	2.15 ± 0.65	2.19 ± 0.65	2.17 ± 0.65	2.12 ± 0.63	2.12 ± 0.66	.561
Kt/V^a^	1.32 ± 0.43	1.33 ± 0.42	1.33 ± 0.40	1.33 ± 0.33	1.30 ± 0.56	.855
Dialysis duration, months	24.44 (12.39, 50.71)	25.95 (11.94, 56.20)	23.98 (12.02, 46.73)	24.80 (13.13, 51.52)	25.43 (12.60, 44.48)	.486
Dietary parameters						
DEI (kcal/kg/day)	29.10 ± 8.33	29.96 ± 8.59	29.86 ± 8.19	28.81 ± 8.38	27.80 ± 8.00	.008
DPI (g/kg/day)	1.07 ± 0.36	1.22 ± 0.40	1.14 ± 0.35	1.03 ± 0.32	0.89 ± 0.28	<.001
DFI (g/kg/day)	0.61 ± 0.31	0.91 ± 0.30	0.68 ± 0.21	0.53 ± 0.18	0.32 ± 0.14	<.001
Fat intake (g/day)	33.42 (16.73)	50.06 (16.55)	37.22 (11.25)	28.74 (9.92)	17.87 (8.31)	<.001
Carbohydrate intake (g/day)	274.96 ± 81.89	239.73 ± 71.16	270.73 ± 69.68	282.18 ± 81.55	306.72 ± 89.41	<.001
Fiber intake (g/day)	8.95 ± 4.04	9.37 ± 4.40	8.95 ± 3.51	8.93 ± 4.24	8.56 ± 3.94	.152
Vegetable intake (g/day)	223.37 ± 124.37	222.09 ± 128.96	228.93 ± 124.23	218.92 ± 116.65	223.49 ± 127.76	.830
Fruit intake (g/day)	114.54 ± 134.15	107.61 ± 126.10	125.74 ± 151.97	110.31 ± 112.21	114.37 ± 142.62	.428

Continuous variables are presented as mean ± standard deviation or median (25th–75th), categorical variables are presented as number (percentage).

^a^Kt/V, Kt showed effective urea clearance and duration of dialysis, and V represents the volume of distribution of urea in the body, calculated as Kt/V = –ln (post-BUN/pre-BUN – 0.008 × t) + (4 – 3.5 × post-BUN/pre-BUN) × UF/post-weight, where *t* is effective dialysis time, BUN is serum blood urea nitrogen and UF is ultrafiltration.

BMI, body mass index.

### Associations between DCIs and the risk of outcomes

During a median follow-up of 45.6 months, all-cause mortality occurred in 352 (33.7%) participants, and 206 (58.5%) deaths were due to CVD.

**Figure 1:**
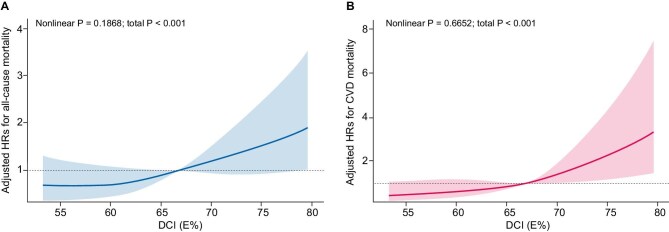
Association between DCI and (**A**) all-cause and (**B**) CVD mortality. Adjusted for dialysis center, age, sex, hypertension, diabetes, history of CVD, smoking status, alcohol consumption, body mass index, Kt/V, dialysis duration, albumin, CRP, total cholesterol, calcium, phosphorus, triglyceride, DEI, DFI, DPI and dietary fiber intake.

The associations between DCI and all-cause and CVD mortality are shown in Fig. 1. In the fully adjusted model (Table 2), compared with those with DCI in the first quartile (<61.5%), a significantly increased risk of all-cause mortality was found in patients in the fourth quartile (≥72.1%; adjusted HR 2.16; 95% CI 1.10, 4.25), although the risk of all-cause mortality was also increased in the second quartile (61.5%–66.7%; adjusted HR 1.27; 95% CI 0.87, 1.87) and the third quartile (66.7%–72.1%; adjusted HR 1.40; 95% CI 0.84, 2.31), but the difference was not statistically significant. Since the HR was similar in the first three quartiles, we grouped these patients together. Compared with those in the first three quartiles (< 72.1%), those with DCIs in the fourth quartile (≥72.1%) consistently presented a significantly increased risk of all-cause mortality (HR 1.46; 95% CI 1.03, 2.07). There was a similar trend in the relationship between DCI and CVD mortality (Table 2).

**Table 2:** Multivariable Cox regression analysis of DCI in relation to mortality.

		Crude		Adjusted^a^	
DCI, E%	Events, *n* (%)	HR (95% CI)	*P*	HR (95% CI)	*P*
All-cause mortality					
<61.5	91 (35.14)	1.0		1.0	
61.5–66.7	85 (32.44)	0.87 (0.65, 1.18)	.376	1.27 (0.87, 1.87)	.220
66.7–72.1	81 (31.15)	0.79 (0.59, 1.07)	.126	1.40 (0.84, 2.31)	.194
≥72.1	95 (36.12)	0.96 (0.72, 1.29)	.807	2.16 (1.10, 4.25)	.026
<72.1	257 (32.91)	1.0		1.0	
≥72.1	95 (36.12)	1.09 (0.86, 1.38)	.471	1.46 (1.03, 2.07)	.034
CVD mortality					
<61.5	51 (19.69)	1.0		1.0	
61.5–66.7	44 (16.79)	0.81 (0.54, 1.21)	.308	1.28 (0.76, 2.14)	.352
66.7–72.1	50 (19.23)	0.88 (0.59, 1.30)	.508	1.73 (0.89, 3.34)	.103
≥72.1	61 (23.19)	1.11 (0.76, 1.61)	.585	2.87 (1.20, 6.87)	.018
<72.1	145 (18.57)	1.0		1.0	
≥72.1	61 (23.19)	1.24 (0.92, 1.68)	.157	1.58 (1.01, 2.49)	.047

^a^Adjusted for dialysis center, age, sex, hypertension, diabetes, history of CVD, smoking status, alcohol consumption, body mass index, Kt/V, dialysis duration, albumin, CRP, total cholesterol, calcium, phosphorus, triglyceride, DEI, DFI, DPI and dietary fiber intake.

**Figure 2:**
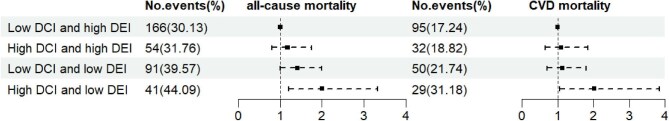
Joint association of DCI and DEI with all-cause mortality and CVD mortality. Adjusted for dialysis center, age, sex, hypertension, diabetes, history of CVD, smoking status, alcohol consumption, body mass index, Kt/V, dialysis duration, albumin, CRP, total cholesterol, calcium, phosphorus, triglyceride, DFI, DPI and dietary fiber intake.

In addition, we analyzed the joint associations of DCI and DEI levels with mortality. Compared with participants with lower DCIs (<72.1%) and higher DEIs (≥25 kcal/kg/day), those with higher DCIs and lower DEIs (<25 kcal/kg/day) were at higher risk of all-cause mortality (HR 2.00; 95% CI 1.20, 3.32) and CVD mortality (HR 2.02; 95% CI 1.06, 3.84). (as shown in Fig. 2). Similarly, the risk of all-cause mortality was also increased in the lower DCIs and lower DEIs group (HR 1.41; 95% CI 1.00, 2.00), reaching borderline significance. Compared with lower DCIs and higher DPIs group, the higher DCIs and higher DPIs group (>1.0 g/kg/day; HR 1.72; 95% CI 1.06, 2.79) had a significantly higher risk of all-cause mortality (shown in Supplementary data, Fig. S2).

### Stratified analysis

**Figure 3:**
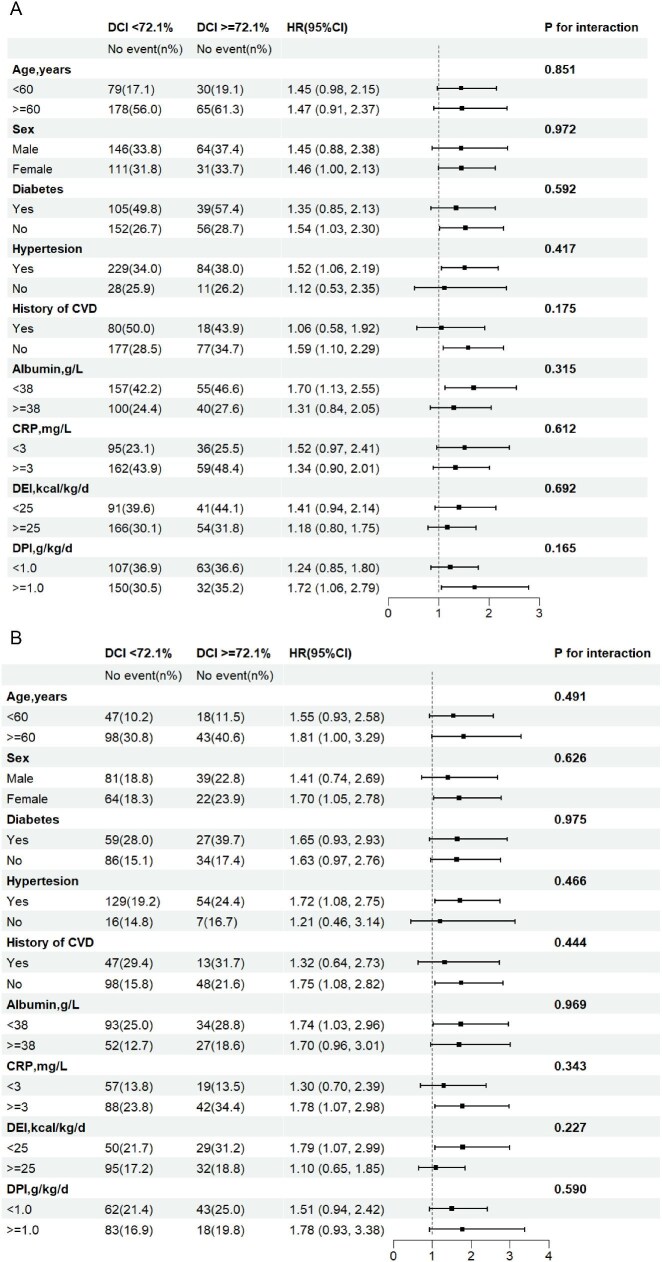
Subgroup analysis of the relationship of DCI with all-cause mortality (**A**) and CVD mortality (**B**). Adjusted for dialysis center, age, sex, hypertension, diabetes, history of CVD, smoking status, alcohol consumption, body mass index, Kt/V, dialysis duration, albumin, CRP, total cholesterol, calcium, phosphorus, triglyceride, DEI, DFI, DPI and dietary fiber intake.

Stratified analyses were conducted to assess the relationship between DCI and the risk of all-cause and CVD mortality in various subgroups. As shown in Fig. 3, stratified analyses showed that the association trend between higher DCI and increased all-cause and CVD mortality was consistent across all subgroups. However, none of these variables significantly modified the correlations between DCI and all-cause or CVD mortality for age (<60 vs ≥60 years), sex (male vs female), diabetes (yes vs no), history of hypertension (yes vs no), history of CVD (yes vs no), serum albumin levels (<38 vs ≥38 g/L), CRP levels (<3 vs ≥3 mg/L), DEI (<25 kcal/kg/day vs ≥25 kcal/kg/day) and DPI (<1.0 g/kg/day vs ≥1.0 kcal/kg/day) (all *P*-values for interactions >.05).

## DISCUSSION

In this multicenter prospective study with a 45.6-month follow-up period, we provide evidence that high DCI levels are associated with a significantly increased risk of all-cause and CVD mortality in patients with MHD. Besides, when DCI and DEI are considered jointly, the patients with higher DCI and lower DEI levels have the highest risk of all-cause and CVD mortality.

Carbohydrates, as the primary source of dietary energy, have garnered increasing attention in recent years. However, the long-term health effects of carbohydrate intake remain poorly understood. In our study, the mean DCI was 66.70% ± 8.22%. A majority of the participants (79.6%) followed a high-carbohydrate diet, deriving at least 60% of their energy from carbohydrates, and more than one-third of the participants obtained more than 70%. This mean DCI in our study was higher than that of MHD patients in Western studies (66.70% vs <56%) [19–21] and was consistent with the DCI in the Chinese general population (67%–69%) [8, 19]. The differences between Chinese and Western DCIs may be related to differences in dietary patterns. Western diets are dominated by meat and dairy products and prefer foods high in fat and energy density, whereas Chinese diets tend to favor high-carbohydrate foods, such as rice, noodles, and noodles steamed with Chinese bread. In addition, people with MHD are often advised to avoid dairy products, fruits, vegetables and whole grains to reduce phosphorus and potassium intake. Moreover, they are encouraged to consume protein-rich foods, especially protein products with high biological value. This resulted in lower intakes of dietary fiber, fruits and vegetables in our study subjects. In addition, to meet energy needs, these individuals tend to increase their intake of carbohydrates and lipids, ultimately leading to high DCIs in our study population.

Our study revealed that DCI levels were positively associated with the risk of all-cause and CVD mortality in patients with MHD, which aligns with findings from several previous studies [6, 8, 9, 20, 21]. Conversely, other studies have indicated that carbohydrate intake is not significantly associated with all-cause mortality [10, 22] or CVD mortality [8–10, 20, 23–25]. This discrepancy may stem from variations in carbohydrate consumption amounts in different populations. One meta-analysis noted that in studies where DCI was not negatively associated with the risk of CVD mortality, DCI was generally lower and predominantly observed in Western populations. In contrast, in cohort studies in Asian countries (such as China and Japan) with an average DCI exceeding 60% and a high intake of refined grains (e.g. white rice), a high DCI was directly associated with the risk of CVD. Additionally, another multinational study reported a nonlinear association between DCI and CVD mortality, revealing a significant increase in CVD mortality when DCI exceeds 60% [11]. Another meta-analysis showed a U-shaped association between carbohydrate intake and all-cause mortality, with the U-shaped curve on the left representing North American and European countries with lower carbohydrate intake and Asian populations with higher carbohydrate intake on the right [26]. A similar controversy has been conducted in CKD populations [7, 12]. However, there is a notable lack of research examining the associations between DCI and all-cause mortality and CVD mortality, especially in patients with MHD. On the basis of the available evidence, this study aims to demonstrate that high DCI increases the risk of all-cause and CVD mortality in the dialysis population.

The exact mechanism by which high DCI leads to a significant increase in all-cause and CVD mortality in MHD patients remains unclear. Several factors may contribute to this phenomenon: most dietary carbohydrates associated with human nutrition have a key ingredient in the monosaccharide d-glucose, which is transported into the blood to stimulate the release of insulin from pancreatic β cells. Glucose is eventually metabolized in a partially insulin-dependent manner. Impaired insulin and/or insulin-like growth factor 1 (IGF-1) signaling may prolong the lifespan of various model organisms. The activation of the corresponding signaling cascade following insulin release may be a key factor in the increased mortality associated with high DCI [27]. Second, excessive carbohydrate intake negatively affects the ability of the ability of the liver to convert it into fat and inhibits the synthesis of beneficial high-density lipoprotein cholesterol. This disruption may lead to dyslipidemia, which is characterized by elevated triglycerides and low cholesterol levels, an elevated apolipoprotein B-to-apolipoprotein A1 ratio and an increase in low-density lipoprotein cholesterol [28, 29], and is associated with increased blood pressure [30]. Collectively, these factors contribute to the development of atherosclerosis and various CVDs. Additionally, refined grains commonly consumed in China, such as rice, white bread and noodles, are often high in starch but deficient in dietary fiber, minerals and phytochemicals. These foods typically have a high glycemic index and glycemic load [31, 32]. Chronic and recurrent postprandial hyperglycemia leads to the overproduction of reactive free radicals and the release of inflammatory cytokines such as interleukin-6 (IL-6), IL-18 and tumor necrosis factor-α (TNF-α), all of which are mediated through the nuclear factor-κB oxidative stress signaling pathway [33, 34]. High intake of these foods is strongly associated with insulin resistance, hyperglycemia, hyperlipidemia and chronic inflammation [11], all of which may contribute to the development of CVD.

In addition, this study jointly evaluated the association between DCI and DEI, as well as DCI and DPI, and all-cause and CVD mortality in patients with MHD. The results of joint analyses were consistent with independent analysis. Patients with high DCIs and low DEIs had a significantly increased risk of all-cause and CVD mortality. Notably, patients with low DCI and low DEI are also at an increased risk of all-cause mortality. This suggests that limiting DCI alone without adequate energy supply may increase the risk of malnutrition and thus lead to poor prognosis. Considering that patients with MHD often have uremia toxin accumulation, nutrient loss during dialysis and chronic inflammation, and are often at high risk of protein-energy consumption, excessive restriction of DCI may lead to insufficient energy intake. At this point, the body may be forced to break down proteins for energy, further exacerbating muscle wasting and inflammation. In addition, a low DCI diet may lead to insufficient intake of micronutrients such as dietary fiber and B vitamins, which may affect intestinal microbiota homeostasis and metabolic regulation. Therefore, when formulating DCI restriction strategies, it is necessary to optimize the levels of DEI and DPI simultaneously to avoid compromising the overall nutritional balance at the cost of reducing carbohydrate intake. To the best of our knowledge, this is the first study to jointly assess the mortality risk of MHD patients in China via DCI and DEI, together with DCI and DPI. These results may provide valuable insights for the dietary management of MHD patients.

Several advantages of our study should be emphasized. First, this is the first cohort study focusing on the associations between DCI and mortality in Chinese MHD patients, adding evidence from a new population to existing population studies. Second, dietary nutrients were collected by 24-h dietary recalls on dialysis and non-dialysis days to minimize interference with dietary intake by dialysis. However, there are several potential limitations of this study that need to be mentioned. First, as an observational study, we cannot make causal statements, and residual confounding by race/ethnicity, genetic factors and other factors not identified by us cannot be ruled out. Because dietary components vary across cultures and geographic regions, the results of this study may not be generalizable to other cultural contexts, and further research is needed to determine the effect of DCI on mortality in patients with MHD in different cultural contexts. Second, dietary intake was only measured at baseline, and dietary patterns may have changed during follow-up. As participants could change their carbohydrate intake during follow-up, any dietary changes that occurred after the baseline assessment would have weakened any observed associations. More frequent measurements would allow for a more accurate assessment of the relationship between DCI and mortality. Third, the study could not distinguish carbohydrates as sugars, starches and food sources (i.e. animal-based vs plant-based), and could not determine the potential impact of carbohydrates. Different types and sources of carbohydrates may have different effects on the prognosis of MHD patients, and further verification is needed. Owing to these limitations, it is essential to confirm our findings with further research.

## CONCLUSIONS

We have shown that high DCI levels were associated with a higher risk of mortality in MHD patients, whereas patients with higher DCI and lower DEI levels had the worst survival outcomes. Therefore, more comprehensive dietary guidelines are needed to facilitate long-term dietary management for MHD patients. The current findings may provide new insights into the dietary management of patients with MHD.

## Supplementary Material

sfaf124_Supplemental_Files

## Data Availability

The data underlying this article will be shared on reasonable request to the corresponding author.
