# Supplementary material for: Association of dietary carbohydrate intake with risk of mortality in maintenance hemodialysis patients: a multicenter prospective cohort study
Source: Clin Kidney J. 2025 Apr 28;18(5):sfaf124. doi: 10.1093/ckj/sfaf124 (PMC12086540; doi:10.1093/ckj/sfaf124)
Supplement: sfaf124_Supplemental_Files [file sfaf124_supplemental_files.zip › 流程图.pptx]

## Slide 1
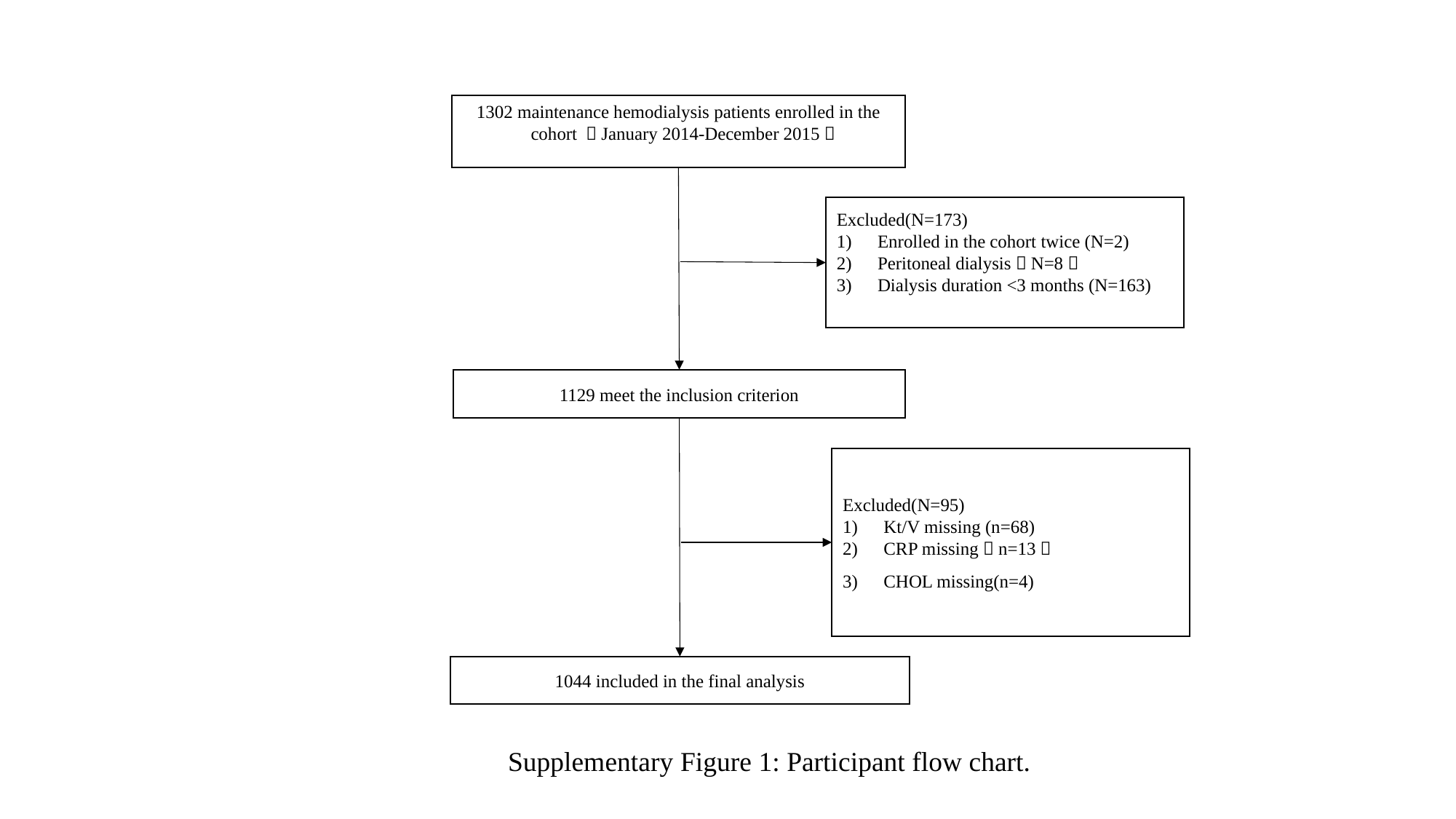

1302 maintenance hemodialysis patients enrolled in the cohort （January 2014-December 2015）
Excluded(N=173)
Enrolled in the cohort twice (N=2)
Peritoneal dialysis（N=8）
Dialysis duration <3 months (N=163)
1129 meet the inclusion criterion
Excluded(N=95)
Kt/V missing (n=68)
CRP missing（n=13）
CHOL missing(n=4)
1044 included in the final analysis
Supplementary Figure 1: Participant flow chart.
