# Supplementary figures and images for: Association of dietary carbohydrate intake with risk of mortality in maintenance hemodialysis patients: a multicenter prospective cohort study
Source: Clin Kidney J. 2025 Apr 28;18(5):sfaf124. doi: 10.1093/ckj/sfaf124 (PMC12086540; doi:10.1093/ckj/sfaf124)

## Slide 1
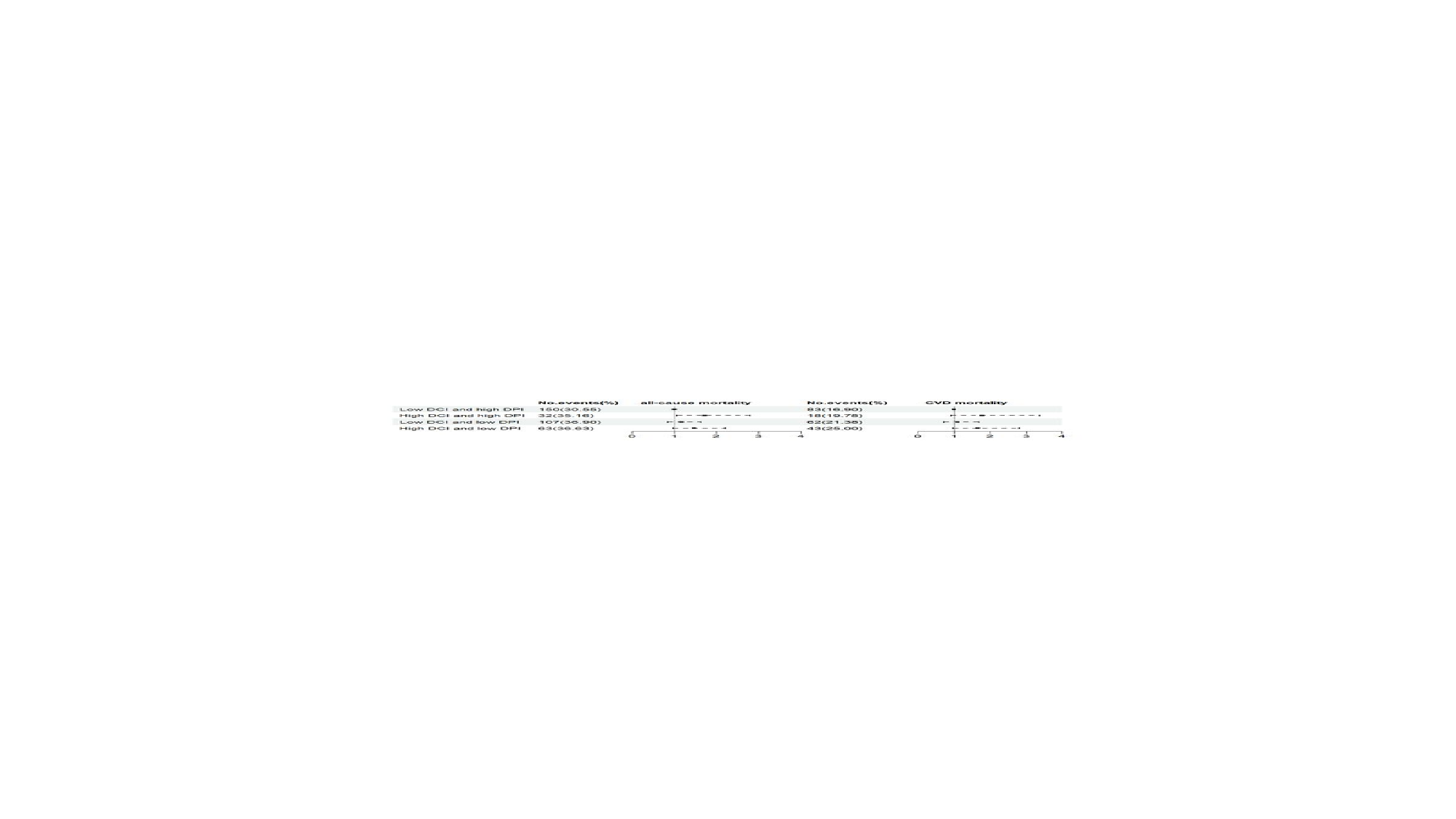

Supplement: sfaf124_Supplemental_Files [file sfaf124_supplemental_files.zip › dci dpi.pptx]
